# Functional Improvements in Parkinson's Disease Following a Randomized Trial of Yoga

**DOI:** 10.1155/2018/8516351

**Published:** 2018-06-03

**Authors:** Marieke Van Puymbroeck, Alysha Walter, Brent L. Hawkins, Julia L. Sharp, Kathleen Woschkolup, Enrique Urrea-Mendoza, Fredy Revilla, Emilie V. Adams, Arlene A. Schmid

**Affiliations:** ^1^Clemson University, Clemson, South Carolina, USA; ^2^Colorado State University, Fort Collins, Colorado, USA; ^3^Bon Secours Neurology, Greenville, South Carolina, USA; ^4^Greenville Health System, Greenville, South Carolina, USA

## Abstract

Individuals with Parkinson's Disease (PD) experience significant limitations in motor function, functional gait, postural stability, and balance. These limitations often lead to higher incidences of falls, which have significant complications for individuals with PD. Yoga may improve these functional deficits in individuals with PD. The objective of this study was to determine changes in motor function, functional gait, postural stability, and balance control for community dwelling individuals with PD. This randomized, wait-list controlled pilot study examined the influence of an 8-week yoga intervention for people with PD who met the following inclusion criteria: endorsing a fear of falling, being able to speak English, scoring 4/6 on the minimental state exam, and being willing to attend the intervention twice weekly for 8-weeks. Participants in the yoga group (n=15) experienced improvements in motor function, postural stability, functional gait, and freezing gait, as well as reductions in fall risk. Participants in the wait-list control (n=12) also significantly improved in postural stability, although their fall risk was not reduced. Individuals in the yoga group significantly reduced their fall risk. An 8-week yoga intervention may reduce fall risk and improve postural stability, and functional and freezing gait in individuals with PD. This clinical trial is registered as protocol record Pro00041068 in clinicaltrials.gov.

## 1. Introduction

In the United States, one million people live with Parkinson's Disease (PD) [1]. Individuals with PD experience substantial functional limitations, specifically in the areas of motor function, functional gait, postural stability, and balance control [2]. Some individuals with PD also experience freezing gait, a phenomenon described as “a brief, episodic absence or marked reduction of forward progression of the feet despite the intention to walk” [3]. Freezing gait can exacerbate mobility impairments and leads to reductions in independence and falls. As gait and balance become more problematic, with or without freezing gait, fall rates increase and individuals develop a fear of falling (FoF), which cascades into reduced participation in life and family roles and diminished recreation participation and is often associated with mental health concerns [4–6]. A recent study found that over 70% of individuals with PD fell within a given year [7]. While there are pharmacological interventions for some symptoms related to PD, often these are insufficient for improving motor function, balance control, postural stability, and freezing of gait [8]. Further, falls lead to serious and costly secondary complications, such as increased mortality, fractures, medical costs, and reduced QoL [9–11].

Consequently, there remains a critical need for innovative programming designed to specifically address fall reductions in people with PD [12]. Balance control is recognized as a complex process that requires an integration of the brain and multiple body systems [13], thus an intervention capable of effectively connecting the brain (mind) and body while targeting multiple body systems has significant potential to improve balance. Yoga is a well-established intervention, known to connect the mind and body, to address multiple systems, and to significantly improve overall balance in people with impaired balance [14–16]. Yoga is a complementary health approach [17]; it is becoming more common for patients to ask their physicians and rehabilitation therapists about complementary and integrative health treatment options [18]. This is often due to concerns regarding costs of conventional medicine, increased side effects from medications, or an attempt to avoid medications or surgery [18]. Integrative health treatment options are frequently lower in cost, have minimal physical and emotional risks, and allow people to take a more active role in their treatment [18].

The practice of Hatha yoga has potential to improve the functional deficits and challenges associated with PD. Hatha yoga is a discipline originating in India and consists of postures (asanas), diaphragmatic breathing (pranayama), and meditation (dhyana) [19]. Hatha yoga is considered the foundation of all other yoga practices; therefore, we refer to Hatha yoga in this intervention simply as ‘yoga' throughout the rest of the text. Although the exact mechanism is unknown, evidence suggests that the combination of postures and breathing is most beneficial when they are utilized together [20] and is considered to produce different effects than simple exercise [21]. Because of the active mind-body component, yoga may be more therapeutic than traditional exercise [22–26]. During yoga, the mind is encouraged to focus on what is occurring in the body and where the body is in space, increasing both awareness and proprioception. The practice of yoga has been associated with improved balance, body alignment, agility, flexibility, strength, overall fitness, endurance, relaxation, and mental and emotional well-being [21, 27–30]. Yoga includes stretching and prolonged physical postures which lengthen major muscle groups and activates the stretch receptors in muscles, ligaments, and joints, leading to improved physical strength and flexibility [27, 29]. The development and testing of yoga-based interventions may significantly improve functional gait, mobility, and balance, and decrease fall rates for people with PD. Thus, the objective of this study was to examine the functional changes in motor function, balance control, postural stability, and freezing of gait that occur following an 8-week yoga intervention for people with PD.

## 2. Methods

This is the primary paper from a randomized, wait-list controlled pilot study of an 8-week yoga intervention designed to increase motor function, balance control, postural stability, and freezing of gait individuals with PD. The study was conducted in partnership between a neurology clinic that employs neurologists trained as Movement Disorder Specialists and a midsize university in the Southeastern United States, and it was approved by the local institutional review boards. All subjects signed the informed consent prior to participation in the study. Recruitment ended after 30 individuals were enrolled in the study. Recruitment ended after 30 individuals were enrolled in the study.

To be included in this study, individuals had to have a diagnosis of PD with a rating of 1.5-3 on the Modified Hoehn and Yahr Scale of Parkinson's Disease Progression [31]; endorse a FoF [32]; be able to stand and walk 10 meters with or without an assistive device; be ≥18 years old; be able to speak English; score ≥4 out of 6 on the short Minimental Status Exam [33]; and be able and willing to attend twice weekly sessions for 8 weeks. Individuals were excluded if they self-reported life expectancy <12 months; identified an inability to attend sessions due to transportation issues; were currently receiving physical therapy or enrolled in an intervention study; or were unable or refused to provide informed consent.

**Figure 1:**
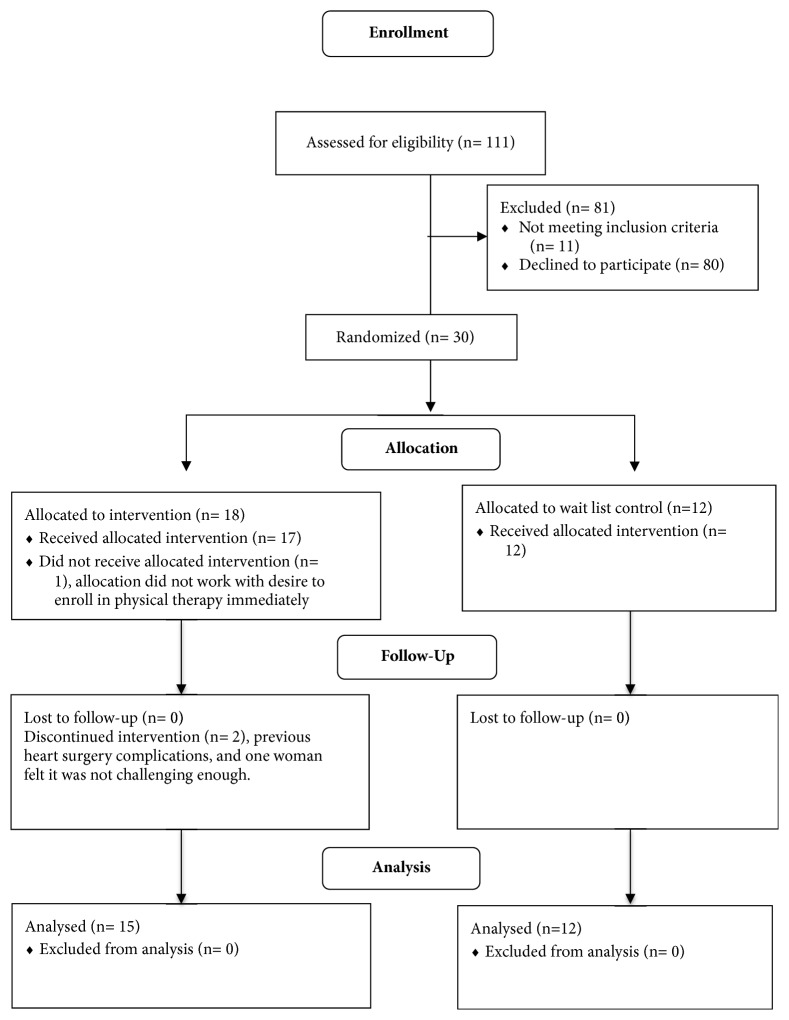
Consort diagram.

Potential participants were identified by the research staff of the neurology clinic, and the project coordinator conducted phone calls to assess interest and potential eligibility. Once individuals were determined to meet eligibility criteria, individuals completed all pretests at the neurology clinic and were subsequently randomly assigned to the experimental (yoga) or wait-list control group in a 2:1 fashion (SAS version 9.3 PROC SURVEYSELECT) by the blinded statistician. The statistician provided group assignments to the principal investigator in sequentially numbered envelopes for distribution following baseline testing. See Figure 1 for the Consort diagram that displays the randomization and group assignment for the study. Each participant was given a $25 gift card for completing the final testing period.

### 2.1. Experimental Group

The participants randomized to the yoga group received a yoga intervention that included controlled dynamic postures connected to specific breathing patterns. The yoga classes were delivered by a certified yoga therapist (C-IAYT) in a standardized progression twice per week for 8 weeks. The yoga intervention included modified yoga postures in sitting, standing, and supine positions as well as pranayama (breath work) and dhyana (meditation). All yoga sessions ended with approximately 10 minutes of relaxation (savasana). Eye pillows, which were given to the participants following conclusion of the study, were used during relaxation in supine resting position. Participants in the group were tested at baseline (T1) and immediately following the intervention (T2).

### 2.2. Wait-List Control

Those assigned to the wait-list control (WLC) were tested at baseline (T1) and 8 (T2) weeks without receiving the yoga intervention and were subsequently offered the yoga intervention, although the data presented here are from the WLC condition (T1 and T2).

### 2.3. Data Collection

Demographics, including age, gender, level of family, and social support, were collected at T1. Other measures were chosen from Parkinson's Evaluation Database to Guide Effectiveness Taskforce recommendations for use in evaluating physical functioning in PD.

The* Movement Disorders Society-Sponsored Revision of the Unified Parkinson's Disease Rating Scale (MDS*-*UPDRS*) motor exam (Part III) was administered primarily by MDS-trained physicians. In a few cases, a physical therapist (PT) that had received extensive training by a MDS-trained study neurologist administered the UPDRS motor exam. Each exam administered by the physical therapist was videotaped, reviewed, and scored by one of the study neurologists. The neurologists and physical therapist were blinded to group assignment. The UPDRS is the gold standard in measuring PD symptoms [34], and the motor exam consists of 18 motor tasks [35].

The* Modified Hoehn and Yahr Scale* describes the symptoms of PD progression in stages ranging from 0 (no signs of disease) to 5 (dependent on a wheelchair or bedridden unless aided) [31]. Progression on Hoehn and Yahr has been correlated with motor decline and worse QoL in individuals with PD and captures the overall pattern of impairments in motor function, regardless of treatment with dopaminergic therapy [36]. This assessment was conducted by MDS-trained study neurologists.

*The Mini-BESTest *is a clinical balance assessment tool that targets 4 balance control systems [5, 37]. The Mini-BESTest contains 14 motor functional tests. The aggregate score is reported here. The Mini-BESTest was administered by a physical therapist who has been trained in the administration of the tool. This assessment has been found to have excellent test-retest reliability and inter/intrarater reliability [38], as well as strong content and construct validity [38, 39] in PD.

*Functional Gait Assessment (FGA) *assesses postural stability during 10 walking tasks [40]. This scale was administered by the PT and contains 10 functional items. Each item is scored on a 4-point scale, where* 0= severe impaired* and* 3= normal ambulation*. The FGA has found to have strong predictive and discriminative validity in a community dwelling population of older adults [41] and in PD [38, 42–44]. Test-retest and inter/intrarater reliability also considered excellent in individuals with PD [38].

*Freezing of Gait Questionnaire (FoG)* is the only validated tool to subjectively assess freezing gait, a common symptom in individuals with PD [45]. This measure assesses frequency of freezing of gait and disturbances in gait using 6 items, where all items are answered on a 5-point scale, where 0= absence of symptoms and 5= most severe stage. Total scores range from 0 to 24, and higher scores indicate greater experience of freezing of gait. The FoG has demonstrated excellent test-retest reliability [46] and excellent internal consistency in PD [45, 46].

### 2.4. Data Analysis

Data were analyzed using SAS version 9.3. Descriptive statistics were used to examine the demographic data. Means, standard error, and effect size (using Cohen's d) were calculated for all measures pre- and postintervention. Effect size is considered small when d≥.20, medium when d ≥.50, and large when ≥.80 [47]. A linear mixed effects model was considered for each scale examining the change from before to after for each group (fixed effect) whereby individuals were treated as random effects. Linear contrasts were used to estimate the change from before to after the study period within each group and to compare intervention and control groups. A significance level of 0.05 was used for all tests of significance. Post hoc correlational analysis was conducted to explore the data further. Hoehn and Yahr scores were examined descriptively due to small sample size.

## 3. Results

Twenty-seven individuals completed the study, with 15 individuals randomized to the yoga group and 12 individuals assigned to the WLC group. Demographics are available in Table 1 and are summarized here. The mean age of participants was 67.75; the majority of participants were male (63%), married (89%), and white (100%), had a college education (60%), and rated their health as excellent or very good (52%).

At the conclusion of the study, across group changes were statistically significant for the FGA (t=2.27, p=0.03), indicating greater functional gait in the experimental group at the end of the yoga intervention, compared with the WLC. This change had a large effect size of d=0.88. All across group changes are displayed for each outcome measure in Table 2.

Within group mean changes are displayed in Table 3. Motor function, as measured by the UPDRS, significantly improved for the yoga group (t(14)=2.97, p=0.0102), but not for the WLC. Improvements in postural stability were noted on the Mini-BESTest (t(14)=-6.01, p<0.0001) in the yoga group and the WLC (t(11=-4.30, p=.0012), while functional gait improved only in the yoga group (t(14)=-6.67, p<0.0001). Freezing of gait significantly improved for the yoga group (t(14)=2.68, p=0.018) but not the WLC.

On the Mini-BESTest, there were statistically significant improvements from T1 to T2 for the yoga group (average change, -5.40; standard error, 3.48; t(14)=-6.01, p<0.0001) and the WLC (-4.00; 3.22; t(11)=-4.30, p<0.0012) which yielded large effects for the yoga group (d=1.55) and the WLC (d=1.24). These data indicate that participants in both groups improved their balance. For the FGA, there were statistically significant improvements for the yoga group (-6.00 (3.49); t(14)=-6.67, p<0.0001) with a large effect size of 1.72, and the WLC (-2.50 (4.54); t(11)=-1.91, p=0.0830) with a medium effect (d=0.55). Significant improvements were also found on the FoG for the yoga group (2.60.; (3.76); t(14) =-2.68, p=0.0179) with a medium effect size of 0.69, while the changes in the WLC were not statistically significant.

At T1, most participants' Modified Hoehn and Yahr score was Stage 2 (n=10 for the yoga group, and n=8 for the WLC), with only one participant in Stage 1.5 for the yoga group, and zero in the WLC. There were four individuals in Stage 3 at T1 in both the yoga and the WLC groups. At T2, five individuals in the yoga group and zero participants in the WLC were classified as Stage 1 or 1.5. There was a reduction in the number of Stage 2 (n=7, Stage 2.5=2) and Stage 3 scores for the yoga group (n=0), while the number of Stage 2 increased at T2 for the WLC (n=6 at 2.0, n=3 at 2.5) and decreased at Stage 3 for the WLC T2 (n=2). A participant in both groups also increased to a Stage 4. See Table 4 for changes in the Hoehn and Yahr Scale.

## 4. Discussion

The purpose of this study was to determine functional changes that occur for individuals with PD following an 8-week yoga intervention. For individuals who completed the yoga intervention, there were significant motor function improvements and a medium effect size on the UPDRS motor subscale. Shulman [48] identified the fact that a minimally clinically important difference (CID) on the UPDRS motor subscale is at least 2.3 points and a moderate CID is 4.5-6.7 points. Thus, the yoga group not only demonstrated a statistically significant improvement but achieved a moderate CID of 6.40 points. Individuals in the WLC group did not demonstrate significant improvement or a minimal CID. Furthermore, in this study there were important level changes in the Hoehn and Yahr ratings. Overall, there was a substantial increase in the numbers of participants in Stage 1 over the eight-week period and notable reductions in Stage 2 and 3. In the WLC, the number of participants in each stage remained fairly static, potentially indicating that motor function and disease progression were improved in the yoga group but not the WLC.

For individuals who completed the yoga intervention, there were balance-related functional improvements and a large effect size on the Mini-BESTest. The WLC also demonstrated an improvement on the Mini-BESTest and a large effect size, though not as large as the yoga group. Duncan et al. [43 identified a score of ≤20/32 on the Mini-BESTest as a cut off score to detect fallers. The data from this study revealed that individuals in the yoga group reduced their fall risk (19.47 T1 vs. 24.87 T2) while individuals in the WLC did not (15.92 T1 vs. 19.92 T2). Thus, it is possible that the individuals in the WLC initiated their own exercise routines which improved balance, but these exercises were not sufficient in effect to reduce fall risk. Another possibility, based on the post hoc correlational analysis, is that during T1, the WLC group took Levodopa closer to test time than the experimental group, thus influencing the scores. The data in this study suggest that the Mini-BESTest demonstrates improvements in balance and reductions in fall risk over time in people with PD following a yoga intervention, an item raised for consideration previously by Boulgarides [49].

Gait-related functional improvements were also demonstrated for both groups on the FGA. Notably, the large effect size for the yoga group (d= 1.72) was more than three times that of the WLC (d=.55), indicating a much greater treatment effect from the yoga intervention. There was a medium effect size (d=.69) and a statistically significant improvement in freezing of gait for individuals in the yoga group. This is an important finding, as freezing of gait has been found to be predictive of falls for individuals who experience it [50]. Freezing of gait did not change in the WLC.

Collectively, the functional improvements in motor function, balance, gait, and freezing of gait indicate that this yoga intervention was successful in reducing fall risk in individuals with PD. This study, conducted by an interdisciplinary research team of neurologists who specialize in movement disorders, recreational therapists, a yoga therapist, occupational therapist, and a PT, indicates that a multidisciplinary approach to fall reduction may be beneficial. Further, yoga has been shown repeatedly to improve balance, but not on measures specifically sensitive to change in PD. The results of this study indicate that even on sensitive measures, yoga may improve functioning in the areas of balance, gait, and freezing of gait. Something that cannot be measured, but it is important also to consider that while some changes may be modest, we are unable to ascertain the slowing of deterioration that may have occurred as a result of this intervention [49].

As with all studies, there are limitations that should be noted. Given the improvements for the WLC in balance and functional gait, it is possible that there was a practice effect. That is, it is possible that those in the WLC learned what the tasks associated with the assessments were and practiced them during the control period. In this study, there may be selection bias, as individuals who opted to participate in the study may be more likely to be compliant with attendance, have an active lifestyle, and have a strong desire to feel in control of their disease. The sample size of this study is small and limits generalizability. Future studies should contain larger sample sizes and consider examining dose of yoga needed to make the optimum changes in functional outcomes for individuals with PD.

Overall, this study demonstrated strong improvements in balance, gait, and freezing of gait for those in the yoga group. Yoga appears to provide a greater reduction in fall risk and a higher magnitude of impact on functional gait than the WLC.

## Figures and Tables

**Table 1:** Demographics.

	Yoga (N=15)	WLC (N=12)	Total
Age (Mean (SD))	65.53 (6.09)	70.5 (4.44)	67.74 (5.89)

Gender (% (N))			
Male	66.67% (10)	58.33% (7)	62.96% (17)
Female	33.33% (5)	41.67% (5)	37.04% (10)

Marital Status (% (n))			
Married	86.67% (13)	91.67% (11)	88.89% (24)
Widowed	0% (0)	8.33% (1)	3.70% (1)
Divorced or separated	13.34% (2)	0% (0)	3.70% (2)

Race (% (n))			
White	100% (15)	100% (12)	100% (27)

Education (% (n))			
High school or less	6.67% (1)	0% (1)	3.70% (2)
College	60% (9)	25% (7)	18.52% (16)
Post-graduate	33.34% (5)	8.33% (4)	7.41% (9)

Overall Health (% (n))			
Excellent or very good	66.67% (10)	0% (4)	3.70% (14)
Good	6.67% (1)	16.67% (2)	11.11% (3)
Fair or poor	26.67% (4)	41.67% (6)	33.33% (10)

**Table 2:** Comparison of average change across groups.

	Group		Average Difference Between Groups (SE); t(df), p	Effect size Cohen's d
	Yoga^+^ (n=15)	Control^+^ (n=12)		
UPDRS	-6.40 (8.35)	-1.17 (13.42)	-5.23 (10.87);	0.48
			t(25)=-1.24, p=0.2255	

MINI	5.40 (3.48)	4.00 (3.22)	1.40 (3.37) t(25)=1.07,	0.42
			p=0.2934	

FGA	6.00 (3.48)	2.50 (4.54)	3.50 (3.99) t(25)=2.27,	0.88
			p=0.0322	

FoG	-2.60 (3.76)	-1.67 (3.11)	-0.93 (3.49) t(25)=-0.69,	0.27
			p=0.4961	

^+^Average change (SD) in score from T1 to T2.

^*∗*^Least squares average difference between yoga and WLC groups in change from T1 to T2 from linear mixed effects analysis.

UPDRS denotes Unified Parkinson's Disease Rating Scale and motor function; MINI denotes Mini-BESTest; FGA denotes Functional Gait Assessment; FoG denotes freezing of gait Questionnaire.

**Table 3:** Comparison of change within yoga and wait-list control groups.

	Yoga (n=15)				Wait List Control (n=12)			
	Mean Pre (SD)	Mean Post (SD)	Average (Post-Pre) Change (SE)^+^; t(df), p	Cohen's d	Mean Pre (SD)	Mean Post (SD)	Average (Post-Pre) Change (SE)^+^; t(df), p	Cohen's d
UPDRS	28.27 (14.89)	21.87 (15.16)	6.40 (8.35); t(14)=2.97, p=0.0102	0.76	31.58 (11.59)	30.42 (11.70)	1.17 (13.42); t(11)=0.30, p=0.7688	0.09

MINI	19.47 (9.70)	24.87 (7.79)	-5.40 (3.48); t(14)=-6.01, p<0.0001	1.55	15.92 (5.76)	19.92 (6.22)	-4.00 (3.22); t(11) =-4.30, p=0.0012	1.24

FGA	14.93 (8.07)	20.93 (8.04)	-6.00 (3.49); t(14)=-6.67, p<0.0001	1.72	15.83 (5.54)	18.33 (7.06)	-2.50 (4.54); t(11)=-1.91, p=0.0830	0.55

FoG	7.60 (6.30)	5.00 (5.44)	2.60 (3.76); t(14)=2.68, p=0.0179	0.69	8.42 (4.81)	6.75 (5.94)	1.67 (3.11); t(11)=1.85, p=0.0907	0.54

^+^Least squares means (standard errors) from linear mixed effects analysis.

UPDRS denotes Unified Parkinson's Disease Rating Scale and motor function; MINI denotes Mini-BESTest;

FGA denotes Functional Gait Assessment; FoG denotes Freezing of Gait Questionnaire

**Table 4:** Modified Hoehn and Yahr staging of the progression of Parkinson's Disease.

Stage	Yoga T1 (n=15)	Yoga T2 (n=15)	WLC T1 (n=12)	WLC T2 (n=12)
1	0	2	0	0

1.5	1	3	0	0

2	10	7	8	6

2.5	0	2	0	3

3	4	0	4	2

4	0	1	0	1

## Data Availability

These data are owned by Clemson University. Access to these data will be considered by the author upon request. She can be reached at mvp@clemson.edu.

## References

[1] Understanding the basics of PD. http://www.apdaparkinson.org/parkinsons-disease/understanding-the-basics/.

[2] Rogers M. W. (1996). Disorders of posture, balance, and gait in Parkinson's disease. *Clinics in Geriatric Medicine*.

[3] Heremans E., Nieuwboer A., Vercruysse S. (2013). Freezing of gait in Parkinson's disease: where are we now?. *Current Neurology and Neuroscience Reports*.

[4] Adkin A. L., Frank J. S., Jog M. S. (2003). Fear of falling and postural control in Parkinson's disease. *Movement Disorders*.

[5] Franchignoni F., Martignoni E., Ferriero G., Pasetti C. (2005). Balance and fear of falling in Parkinson's disease. *Parkinsonism & Related Disorders*.

[6] Mak M. K. Y., Pang M. Y. C. (2009). Fear of falling is independently associated with recurrent falls in patients with Parkinson's disease: a 1-year prospective study. *Journal of Neurology*.

[7] Bloem B. R., Hausdorff J. M., Visser J. E., Giladi N. (2004). Falls and freezing of Gait in Parkinson's disease: a review of two interconnected, episodic phenomena. *Movement Disorders*.

[8] Bloem B. R., Grimbergen Y. A. M., Cramer M., Willemsen M., Zwinderman A. H. (2001). Prospective assessment of falls in Parkinson's disease. *Journal of Neurology*.

[9] Bennett D. A., Beckett L. A., Murray A. M. (1996). Prevalence of Parkinsonian signs and associated mortality in a community population of older people. *The New England Journal of Medicine*.

[10] De Boer A. G. E. M., Wijker W., Speelman J. D., De Haes J. C. J. M. (1996). Quality of life in patients with Parkinson's disease: development of a questionnaire. *Journal of Neurology, Neurosurgery & Psychiatry*.

[11] Ebmeier K. P., Calder S. A., Crawford J. R., Stewart L., Besson J. A. O. (1990). Mortality and Causes of Death in Idiopathic Parkinson's Disease: Results from the Aberdeen Whole Population Study. *Scottish Medical Journal*.

[12] Ashburn A., Fazakarley L., Ballinger C., Pickering R., McLellan L. D., Fitton C. (2007). A randomised controlled trial of a home based exercise programme to reduce the risk of falling among people with Parkinson's disease. *Journal of Neurology, Neurosurgery & Psychiatry*.

[13] Rogers M. W., Martinez K. M., Stein J, Harvey R. L., Macko R. F., Winstein C., Zorowitz R. D. (2009). Recovery and Rehabiliation of Standing Balance after Stroke. *Stroke Recovery and Rehabilitation*.

[14] Schmid A. A., Van Puymbroeck M., Altenburger P. A. (2012). Poststroke balance improves with yoga: a pilot study. *Stroke*.

[15] Schmid A. A., van Puymbroeck M., Koceja D. M. (2010). Effect of a 12-week yoga intervention on fear of falling and balance in older adults: a pilot study. *Archives of Physical Medicine and Rehabilitation*.

[16] Garrett R., Immink M. A., Hillier S. (2011). Becoming connected: The lived experience of yoga participation after stroke. *Disability and Rehabilitation*.

[17] Complementary, Alternative, or Integrative Health: What’s In a Name?. https://nccih.nih.gov/health/integrative-health.

[18] Wahbeh H., Elsas S. M., Oken B. S. (2008). Mind-body interventions: applications in neurology. *Neurology*.

[19] Yoga. https://nccih.nih.gov/health/yoga.

[20] Kirkwood G., Rampes H., Tuffrey V., Richardson J., Pilkington K. (2005). Yoga for anxiety: a systematic review of the research evidence. *British Journal of Sports Medicine*.

[21] Cameron M., Snyder M., Lindquist R. (2006). Yoga. *Complementary/Alternative Therapies in Nursing*.

[22] Berger B. G., Owen D. R. (1988). Stress reduction and mood enhancement in four exercise modes: swimming, body conditioning, hatha yoga, and fencing. *Research Quarterly for Exercise and Sport*.

[23] Brown D. R., Wang Y., Ward A. (1995). Chronic psychological effects of exercise and exercise plus cognitive strategies. *Medicine & Science in Sports & Exercise*.

[24] Chan A. S., Ho Y.-C., Cheung M.-C., Albert M. S., Chiu H. F. K., Lam L. C. W. (2005). Association between mind-body and cardiovascular exercises and memory in older adults. *Journal of the American Geriatrics Society*.

[25] Berger B. G., Owen D. R. (1992). Mood alteration with yoga and swimming: aerobic exercise may not be necessary. *Perceptual and Motor Skills*.

[26] Van Puymbroeck M., Hsieh P. C., Pernell D. (2008). The influence of mindfulness-based stress reduction and walking on the psychological well-being of female informal caregivers. *American Journal of Recreation Therapy*.

[27] Luskin F. M., Newell K. A., Griffith M. (2000). A review of mind/body therapies in the treatment of musculoskeletal disorders with implications for the elderly. *Alternative Therapies in Health and Medicine*.

[28] Bal B. S., Kaur P. J. (2009). Effects of selected asanas in hatha yoga on agility and flexibility level. *Journal of Sport and Health Research*.

[29] Tran M. D., Holly R. G., Lashbrook J., Amsterdam E. A. (2001). Effects of hatha yoga practice on the health-related aspects of physical fitness. *Preventive Cardiology*.

[30] Van Puymbroeck M., Payne L. L., Hsieh P.-C. (2007). A phase I feasibility study of yoga on the physical health and coping of informal caregivers. *Evidence-Based Complementary and Alternative Medicine*.

[31] Goetz C. G., Poewe W., Rascol O. (2004). Movement disorder society task force report on the Hoehn and Yahr staging scale: status and recommendations. *Movement Disorders*.

[32] Walker J. E., Howland J. (1991). Falls and fear of falling among elderly persons living in the community: occupational therapy interventions.. *The American journal of occupational therapy. : official publication of the American Occupational Therapy Association*.

[33] Callahan C. M., Unverzagt F. W., Hui S. L., Perkins A. J., Hendrie H. C. (2002). Six-item screener to identify cognitive impairment among potential subjects for clinical research. *Medical Care*.

[34] Ebersbach G., Baas H., Csoti I., Müngersdorf M., Deuschl G. (2006). Scales in Parkinson's disease. *Journal of Neurology*.

[35] Goetz C. G., Tilley B. C., Shaftman S. R. (2008). Movement Disorder Society-Sponsored Revision of the Unified Parkinson's Disease Rating Scale (MDS-UPDRS): scale presentation and clinimetric testing results. *Movement Disorders*.

[36] Bhidayasiri R., Tarsy D. (2012). Movement Disorders: A Video Atlas. *Current Clinical Neurology*.

[37] Franchignoni F., Horak F., Godi M., Nardone A., Giordano A. (2010). Using psychometric techniques to improve the balance evaluation systems test: the mini-bestest. *Journal of Rehabilitation Medicine*.

[38] Leddy A. L., Crowner B. E., Earhart G. M. (2011). Functional gait assessment and balance evaluation system test: reliability, validity, sensitivity, and specificity for identifying individuals with parkinson disease who fall. *Physical Therapy in Sport*.

[39] King L. A., Priest K. C., Salarian A., Pierce D., Horak F. B. (2012). Comparing the Mini-BESTest with the Berg Balance Scale to evaluate balance disorders in Parkinson's disease. *Parkinson’s Disease*.

[40] Wrisley D. M., Marchetti G. F., Kuharsky D. K., Whitney S. L. (2004). Reliability, internal consistency, and validity of data obtained with the functional gait assessment. *Physical Therapy in Sport*.

[41] Wrisley D. M., Kumar N. A. (2010). Functional gait assessment: concurrent, discriminative, and predictive validity in community-dwelling older adults. *Physical Therapy in Sport*.

[42] Duncan R. P., Leddy A. L., Cavanaugh J. T. (2012). Accuracy of fall prediction in parkinson disease: six-month and 12-month prospective analyses. *Parkinson’s Disease*.

[43] Foreman K. B., Addison O., Kim H. S., Dibble L. E. (2011). Testing balance and fall risk in persons with Parkinson disease, an argument for ecologically valid testing. *Parkinsonism & Related Disorders*.

[44] Foreman K. B., Wisted C., Addison O., Marcus R. L., Lastayo P. C., Dibble L. E. (2012). Improved dynamic postural task performance without improvements in postural responses: The blessing and the curse of dopamine replacement. *Parkinson’s Disease*.

[45] Giladi N., Shabtai H., Simon E. S., Biran S., Tal J., Korczyn A. D. (2000). Construction of freezing of gait questionnaire for patients with Parkinsonism. *Parkinsonism & Related Disorders*.

[46] Giladi N., Tal J., Azulay T. (2009). Validation of the freezing of gait questionnaire in patients with Parkinson's disease. *Movement Disorders*.

[47] Cohen J. (1988). *Statistical power analysis for the behavioral sciences*.

[48] Shulman L. M., Gruber-Baldini A. L., Anderson K. E., Fishman P. S., Reich S. G., Weiner W. J. (2010). The clinically important difference on the unified parkinson's disease rating scale. *JAMA Neurology*.

[49] Boulgarides L. K., Barakatt E., Coleman-Salgado B. (2014). Measuring the effect of an eight-week adaptive yoga program on the physical and psychological status of individuals with Parkinson's disease. A pilot study. *International Journal of Yoga Therapy*.

[50] Latt M. D., Lord S. R., Morris J. G. L., Fung V. S. C. (2009). Clinical and physiological assessments for elucidating falls risk in Parkinson's disease. *Movement Disorders*.

