# Corrigendum to “Functional Improvements in Parkinson's Disease Following a Randomized Trial of Yoga”

**DOI:** 10.1155/2018/4523743

**Published:** 2018-09-06

**Authors:** Marieke Van Puymbroeck, Alysha A. Walter, Brent L. Hawkins, Julia L. Sharp, Kathleen Woschkolup, Enrique Urrea-Mendoza, Fredy Revilla, Emilie V. Adams, Arlene A. Schmid

**Affiliations:** ^1^Clemson University, Clemson, South Carolina, USA; ^2^Colorado State University, Fort Collins, Colorado, USA; ^3^Bon Secours Neurology, Greenville, South Carolina, USA; ^4^Greenville Health System, Greenville, South Carolina, USA

In the article titled “Functional Improvements in Parkinson's Disease Following a Randomized Trial of Yoga” [1], the name of second author was given incorrectly as Alysha Walter. The author's name should have been written as Alysha A. Walter. The revised authors' list is shown above.
